# Recognising and addressing social determinants of health: an important step toward centring equity in cardiovascular care

**DOI:** 10.1007/s12471-024-01857-9

**Published:** 2024-02-16

**Authors:** Bigina N. R. Ginos, Maryam Kavousi

**Affiliations:** Department of Epidemiology, Erasmus MC University Medical Center, Rotterdam, The Netherlands

Despite the strong decline in mortality due to myocardial infarction throughout the twentieth century, coronary heart disease remains the largest contributor to disability-adjusted life years in the Netherlands [1]. In both the Dutch and global context, individuals with low socioeconomic status (SES) bear a disproportionate burden of cardiovascular disease (CVD), as evidenced by higher event and mortality rates [2]. Moreover, cardiovascular risk factors have been shown to cluster around low SES and other psychosocial risk factors [3], impacting not only the likelihood of developing CVD but also the efficacy of CVD care [2, 3]. These CVD risk clusters may in part be explained by limitations of current individual-level efforts to manage clinical predictors of CVD (e.g. blood pressure, lipid levels and glucose levels), which are reliant on psychological and behavioural factors [3] that are most likely subject to social stratification. Barriers to mitigating psychosocial risk factors and implementing behavioural changes across SES are found in upstream determinants of health (e.g. housing security, neighbourhood resources, social cohesion, health care access) [4]. These overarching social domains shape the experiences of an individual as well as those of the community.

Among 7264 patients treated in three Dutch percutaneous coronary intervention (PCI) centres, Hilt et al. [5] evaluated whether the type of revascularisation after myocardial infarction differed between patients in the highest (*n* = 1040) and lowest (*n* = 1639) quartile of SES. They retrieved SES scores for each residential postal code, which were calculated by the Dutch government based on yearly income, employment status and education. Information on treatment was obtained from the claims data. Patients from the lowest SES group underwent coronary artery bypass grafting (CABG) more frequently and PCI less frequently compared to patients in the highest SES group. The trends were independent of myocardial infarction type (i.e. ST-elevation myocardial infarction (STEMI) or non-ST-elevation myocardial infarction (NSTEMI)). Hilt et al. interpreted these differences in treatment distribution to be a potential indicator that patients from low SES areas present more frequently with complex coronary lesions, requiring more invasive treatment. The authors hypothesised that this could be due to an increased burden of cardiovascular risk factors, such as smoking, hypertension and diabetes mellitus, in the low SES group. One year after the initial diagnosis of STEMI or non-STEMI, a greater proportion of patients in the lowest SES category than that in the highest SES category received optimal medical treatment, defined as a combination of aspirin, P2Y12 inhibitor, statin, beta blocker and angiotensin-converting enzyme/angiotensin II inhibitor. This trend was limited to patients who suffered a STEMI. The authors assigned these interesting results to a possible unwillingness of highly educated individuals to use medication. They speculated, on the other hand, that lower medication use could also be an indicator of better health in the high SES group. Of note is that the overall number of patients receiving optimal medical treatment was low across all groups (STEMI: 52% in lowest SES and 45% in highest SES; NSTEMI: 39% in lowest SES and 40% in highest SES). Hilt et al. correctly recommend that such findings warrant development of strategies to improve medication adherence after revascularisation, regardless of SES.

While the study was limited to a specific patient population (patients who underwent PCI or CABG after myocardial infarction) and confined areas (Leeuwarden, Alkmaar and Leiden), the results of Hilt et al. [5] offer a segue into a broader discussion on social determinants of cardiovascular health. The authors point out the limitations of using postal code area SES, as it may lead to misclassification when individuals with high SES live in low SES areas or vice versa. However, linking area-based SES variables to patient data may help identify upstream barriers and shape how to approach primary prevention strategies. Previous studies in Europe [6] and North America [7] described independent associations of neighbourhood SES with coronary heart disease risk and case fatality that remained after adjustment for individual-level SES. The impact of living environment on cardiovascular health may even stretch across the life course. In a recent study, larger hazard ratios for CVD were observed in individuals who resided in areas with the lowest SES from young adulthood and throughout older adulthood [8]. Furthermore, a large longitudinal European and Australian multi-cohort study described both between- and within-neighbourhood mortality risk gradients, in which residents with low SES in low SES neighbourhoods were at greatest risk [9]. These findings underline the necessity of evaluating social determinants of health both at an individual and systemic level.

The method presented by Hilt et al. [5], which utilises various data sources, constitutes an additional tool that can help in understanding the needs of communities and achieving health equity. To further build on this, a mixed-methods approach may be employed. First, geocoding residential addresses will allow linkage to a wide range of publicly available governmental ‘living environment’ maps, such as social cohesion, green space and walkability. Next, qualitative data collection will provide insights into the systemic context and experienced barriers to optimal cardiovascular health. Finally, electronic health record data may be used to identify clinical and intermediate health outcomes, which will be quantitatively analysed in relation to living environment characteristics and intersecting barriers. Local policymakers and healthcare facilities should aim to work together with patients and residents to address the identified local needs.
